# Bio-Composite Progress (Part A): Advances in Mycelium-Based Composites as Sustainable Alternatives to Plastic and Foam Packaging

**DOI:** 10.3390/polym18192340

**Published:** 2026-09-25

**Authors:** Worawoot Aiduang, Kritsana Jatuwong, Didsanutda Gonkhom, Orlavanh Xayyavong, Saisamorn Lumyong

**Affiliations:** 1 Office of Research Administration, Chiang Mai University, Chiang Mai 50200, Thailand; worawoot.aiduang@cmu.ac.th (W.A.); aew.kritsana@gmail.com (K.J.); 2 Department of Biology, Faculty of Science, Chiang Mai University, Chiang Mai 50200, Thailand; didsanutda8725@gmail.com (D.G.); xorlavanh@yahoo.com (O.X.); 3 Department of Biology, Faculty of Science, Champasack University, Pakse 16010, Laos; 4 Center of Excellence in Microbial Diversity and Sustainable Utilization, Chiang Mai University, Chiang Mai 50200, Thailand; 5 Academy of Science, The Royal Society of Thailand, Bangkok 10300, Thailand

**Keywords:** biodegradable materials, biomass valorization, eco-friendly packaging, mycelium-based technologies, SDGs 9

## Abstract

The increasing environmental burden of petroleum-based plastic and foam packaging has accelerated the search for sustainable bio-based alternatives. This study developed mycelium-based cushioning packaging (MBCP) using *Lentinus sajor-caju* cultivated on four abundant biomass residues (corn husk, rice straw, sawdust, and sugarcane leaves) and comprehensively evaluated their physical, mechanical, microstructural, biodegradation, phytotoxicity, and cushioning performances. Substrate selection significantly influenced composite properties. Sawdust-based MBCP exhibited the highest density (314.79 kg/m^3^), superior compressive strength (0.91 MPa), and the lowest water absorption and volumetric swelling, indicating excellent structural stability under humid conditions. In contrast, corn husk- and sugarcane leaf-based composites developed superiorly porous structures that enhanced impact energy dissipation, with corn husk demonstrating better cushioning performance in free-fall drop tests, outperforming conventional air bubble wrap and expanded polyethylene foam in preventing secondary damage to glass bottles. Scanning electron microscopy confirmed that variations in biomass composition affected mycelial formation, pore distribution, and network density, directly influencing mechanical behavior and impact resistance. Soil burial experiments revealed progressive biodegradation, with corn husk- and sugarcane leaf-based MBCP achieving over 60% degradation within 90 days, while phytotoxicity assays revealed no inhibitory effects on seed germination and even promoted early seedling growth in several vegetable species, supporting safe environmental disposal and potential secondary use as biodegradable planters. Collectively, these findings demonstrate that biomass residues can be strategically selected to improve the functional performance of MBCP by offering a compromise between biodegradability, mechanical durability, and cushioning efficiency. The developed MBCP represents a promising renewable alternative to conventional petroleum-derived plastic and foam packaging, supporting circular bioeconomy strategies through waste valorization and environmentally responsible packaging solutions.

## 1. Introduction

The rapid expansion of global buying and e-commerce has significantly increased the demand for packaging materials, particularly for fragile and high-value products such as glass bottles, cosmetics, pharmaceuticals, and specialty food and beverage products [1,2]. These applications require packaging materials that can withstand handling, vibration, impact, and compression while maintaining low weight and dimensional stability. Conventional packaging materials, especially plastics and foams such as air bubble wrap, expanded polyethylene foam (EPE), and expanded polystyrene (EPS), are widely used due to their excellent cushioning performance, easy access, and low cost [3,4]. However, these petroleum-based materials pose serious environmental challenges because of their non-biodegradability, high carbon footprint, and long-term durability in ecosystems [5]. Consequently, there is an urgent need to develop sustainable, biodegradable, and high-performance alternatives that can effectively replace synthetic packaging materials without compromising protective functionality [6].

In this context, bio-composites derived from fungal mycelium and lignocellulosic biomass residues have emerged as an interesting group of sustainable materials [7]. Mycelium-based composites (MBCs) are formed through the natural ability of fungal hyphae to bind and interlock agricultural residue particles into lightweight, well-formed, and biodegradable materials [8]. These technologies not only provide an environmentally friendly alternative to plastic and foam packaging but also contribute to biomass valorization by transforming agricultural waste into value-added products. This approach aligns strongly with circular economy principles and supports the achievement of sustainable development goals (SDGs), particularly SDG 8 (decent work and economic growth) and SDG 9 (industry, innovation, and infrastructure) [9,10].

Among various fungal species, *Lentinus sajor-caju* has demonstrated strong potential for MBC development in our previous studies, owing to its rapid colonization, dense mycelial network formation, and favorable mechanical performance in composite structures [11,12,13,14]. When combined with multiple types of agricultural residues such as corn husk, rice straw, and rubber tree sawdust, this fungal species can effectively convert low-value biomass into functional materials [11]. In addition, the performance of such composites is further influenced by substrate formulation and processing conditions, all of which play critical roles in determining the structural integrity and improving functional performance of the final products [13].

For packaging applications, particularly cushioning mechanisms for high-value bottle products, material performance requirements extend beyond biodegradability to include mechanical strength, energy absorption, dimensional stability, and environmentally friendly properties [8,15,16]. Therefore, the development of mycelium-based cushioning packaging (MBCP) requires not only optimized biological growth but also carefully selected substrate characteristics and fabrication techniques, including mold design, compression processing, and controlled formation. These factors collectively determine the quality, consistency, and scaling ability of the final products [9].

Despite recent advances in MBCP technology and the growing body of research evaluating their performance under practical conditions [7,8], further extensive investigation is needed to clarify their effectiveness as protective packaging materials under application-relevant handling, loading, and impact conditions. Comprehensive comparative studies integrating physical and structural properties, mechanical performance, biodegradability, phytotoxicity, and actual packaging protection performance across different biomass substrates are still limited. Such integrated evaluation is essential for identifying the relationships between substrate composition, material characteristics, and functional performance, as well as for determining their commercial potential. Moreover, direct comparison with conventional packaging materials is necessary to establish the functional competitiveness and economic feasibility of MBCP as sustainable alternatives for protective packaging applications. In particular, the substantially higher mass of MBCP compared with lightweight conventional materials, such as air bubble wrap and EPE foam, represents an important practical consideration because increased packaging weight can raise transportation costs and transportation-related carbon emissions, particularly over long distances and in air freight. Therefore, the environmental and economic advantages of MBCP should be evaluated in relation to its transportation requirements and intended distribution routes rather than solely on the basis of material biodegradability or waste valorization. Thus, this study aimed to develop and evaluate the overall potential of MBCP derived from agricultural and agro-industrial residues. The research focuses on determining key processes, covering inoculum preparation, substrate formulation, and fabrication techniques, while thoroughly examining the resulting material attributes to enhance performance for protective bottle packaging applications. Through integrated biological, material, and engineering approaches, this work seeks to advance sustainable packaging innovation while promoting resource efficiency, waste valorization, and green material design.

## 2. Materials and Methods

### 2.1. Preparation of Fungal Mycelium and Production of Inoculum

The fungal strain used in this study, *Lentinus sajor-caju,* was selected based on its high potential demonstrated in our previous research [11]. This species has shown strong performance in the production of MBCs, exhibiting favorable properties and potential as an alternative to synthetic foam materials. Prior to use, the fungal culture was cultivated on potato dextrose agar (PDA; Conda, Madrid, Spain) and incubated at 30 °C for 7 days to obtain actively growing mycelium [17].

For MBCP specimens’ formation, the mycelium was prepared as an inoculum using sorghum grains as the substrate, following our previously established method. The grains were thoroughly washed and boiled for 20 min before transferring 150 g portions into glass bottles. Each bottle was sealed with cotton wool, covered with aluminum foil, and sterilized by autoclaving at 121 °C for 20 min. After cooling to room temperature, five mycelial plugs (5 mm in diameter) from actively growing PDA cultures were aseptically inoculated into each bottle. The inoculated bottles were then incubated at 30 °C for two weeks to ensure complete colonization of the sorghum grains by the mycelium [18].

### 2.2. Collection and Preparation of Biomass Residues

Four types of agricultural and agro-industrial residues, namely corn husk, rice straw, rubber tree sawdust, and sugarcane leaves, were selected and used as the primary substrates in this study. These residues were collected from agricultural areas, including corn and rice fields, as well as from a sawmill located in Chiang Mai Province, Thailand.

The corn husk, rice straw, and sugarcane leaves were processed using a wood chipper and subsequently sieved to obtain comparable particle sizes ranging from 5–10 mm for experimental use. Meanwhile, the rubber tree sawdust was sieved through a 10 mm mesh to remove large pieces and unacceptable residues. Prior to composite formation, all selected substrates were dried in a hot-air oven at 60 °C until completely dry [11].

### 2.3. Substrate Formulation for Mycelial Growth and Mold Preparation

#### 2.3.1. Preparation of Substrate Mixtures and Mycelial Inoculation

Substrate preparation and mixing ratios were conducted according to the method described in our previous study [11]. The substrate mixtures were supplemented with 5% rice bran, 1% calcium carbonate, 2% calcium sulfate, and 0.2% sodium sulfate on a dry weight basis. The moisture content of each mixture was then adjusted to approximately 60% by adding distilled water. Approximately 500 g of the prepared substrate mixture was filled into polypropylene (PP) plastic bags fitted with PP pipe rings and sealed using cotton-plastic plugs. The bags were subsequently sterilized by autoclaving at 121 °C for 60 min. After sterilization, the substrates were allowed to cool to room temperature for 24 h. Following cooling, approximately 5 g of the prepared mycelial inoculum was aseptically inoculated onto the surface of the substrate in each bag. The inoculated bags were then incubated at 30 °C under dark conditions. Complete mycelial colonization of the substrates was observed after 14–21 days of incubation, after which the colonized substrates were considered ready for the MBCP formation process.

#### 2.3.2. Design and Sterilization of Molds

The molds used for fabricating MBCP were modified from the design reported by Jiao et al. [19]. The molds were designed from transparent acrylic cylinders measuring 64 mm in diameter and 85 mm in height. The inner core component was fabricated from a nylon superlene solid rod with dimensions of 45 mm in diameter and 75 mm in height and was fixed using transparent acrylic rings. The mold cover was constructed from clear acrylic sheets cut into circular shapes with dimensions of 63 mm in diameter and 15 mm in thickness. Prior to use, all mold components were sterilized by immersion in a 2% sodium hypochlorite solution (Hichlor; Chiang Mai, Thailand) for 5 min, followed by two rinses with sterile distilled water. The molds were then air-dried under aseptic conditions inside a laminar flow cabinet to minimize the risk of contamination before use in the experiments [13].

### 2.4. Formation of Mycelium-Based Cushioning Specimens

The MBCP was fabricated following the method and conditions described in our previous study [11]. Substrates fully colonized by fungal mycelium and ready for MBCP fabrication were reground into smaller pieces before being transferred into the designed molds. The filled molds were compressed using a unidirectional cold press machine (Shop Press ZX0901E-1; OMASTAR, New Taipei, Taiwan) at 0.5 MPa for 5–10 min and subsequently incubated at 30 °C. After 3 days of incubation, the MBCP was carefully removed from the molds and transferred to separate plastic containers for an additional 3 days to allow complete mycelial growth over the surfaces previously in contact with the molds. The obtained MBCP was subsequently measured and its total volume was recorded prior to drying. The samples were then dried in a hot-air oven at 70 °C for 24–48 h until a constant weight was achieved. Finally, the dried MBCP was stored in desiccators before further experimental analyses.

### 2.5. Characterization of Mycelium-Based Cushioning Specimens

#### 2.5.1. Evaluation of Physical Properties

The shrinkage of the developed MBCP was determined based on the difference between wet and dry volumes following the method adapted from Elsacker et al. [20]. Shrinkage was expressed as a percentage using the following equation: shrinkage percentage (%) = [(*V*_1_ − *V*_2_)/*V*_1_] × 100 (where *V*_1_ represents the wet volume of the sample and *V*_2_ represents the dry volume of the sample).

The MBCP was cut into specimens measuring 20 × 30 mm. Sample thickness was measured using a digital vernier caliper, while the specimen volume was calculated from the measured area and thickness. The samples were subsequently dried in a hot-air oven at 70 °C for 24 h until a constant weight was achieved. The dry weight of each specimen was then measured using a digital laboratory balance with a precision of 0.0001 g. Density was calculated according to the standard method based on the mass-to-volume ratio of the specimens [21].

Water absorption and volumetric swelling were evaluated according to ASTM D1037:2020, following the modified method of Zimele et al. [22]. Prior to testing, the MBCP was oven-dried at 70 °C until a constant mass was achieved and then cooled in a desiccator for 24 h. The initial dry mass of each specimen was measured using a digital analytical balance (±0.01 g) before immersion in deionized water. The specimens were immersed for 24 h, and their masses were recorded after 2 and 24 h. Water absorption was calculated using the following equation: Mass increasing (%) = [(*D*_1_ − *D*_2_)/*D*_2_] × 100, where *D*_1_ is the wet mass and *D*_2_ is the dry mass of the sample. In addition, the volumetric swelling was determined after 24 h of immersion in distilled water by calculating the percentage increase in specimen volume relative to its initial volume.

#### 2.5.2. Observation of Outer and Inner Surface Microstructures of Developed MBCP

The distribution and coverage of fungal mycelium on the surface microstructures of MBCP can significantly influence their overall properties and performance. In this study, all dried MBCP samples were cut into small square sections (5 mm) from both the outer and inner surfaces using a scalpel to investigate their microstructural characteristics. The prepared samples were mounted onto 10 mm^2^ stub adapters using 5 mm double-sided carbon tape and coated with gold for 2 min under high-vacuum conditions. Subsequently, the samples were examined and photographed using a scanning electron microscope (SEM JSM-IT300, JEOL, Tokyo, Japan) operated at an accelerating voltage of 15 kV. SEM analysis was conducted at the Science and Technology Service Center, Faculty of Science, Chiang Mai University, Chiang Mai, Thailand. The outer and inner surface characteristics of all developed MBCP samples were then comparatively evaluated among the different treatments to determine variations in mycelial network formation and structural morphology.

#### 2.5.3. Measurement of Mechanical Properties

In this study, the mechanical property analysis was conducted using standardized methods adapted for comparable composite container specimens to obtain relevant and applicable results. The compressive strength of the MBCP was determined following ASTM D695-15 using a modified method described by Choowang et al. [23]. Tests were conducted using a Hounsfield-H10Ks universal testing machine (Tinius Olsen, New York, NY, USA) equipped with a 10 kN load frame and a 1 kN load cell under ambient conditions (25 °C and 40–50% relative humidity). Compression testing was performed at a controlled displacement rate of 6 mm/min. The obtained load–displacement data were converted into stress–strain curves using the following equations: compressive stress (σ) = F/A and strain (ε) = ΔL/L_0_, where F represents the compressive force (N), A is the original cross-sectional area of the specimen (mm^2^), ΔL is the displacement during loading (mm), and L_0_ is the original height of the specimen (mm). Compression strength values were reported in MPa.

### 2.6. Assessment of Biodegradability and Phytotoxicity Using Plant-Based Assays

#### 2.6.1. Soil Burial Degradation Test

The biodegradability of the MBC specimens was evaluated using a soil burial method adapted from ISO 846:2000. The specimens used in this experiment were cylindrical MBC specimens (approximately 8.2 cm in diameter × 1.0 cm in height) prepared in plastic molds following the procedure described in our previous study [11] under the same manufacturing conditions described in Section 2.4. Commercial potting soil was used as the degradation medium. Before testing, the soil was sieved through a 2-mm mesh to obtain a comparable particle size. Prior to burial, the specimens were oven-dried at 70 °C to a constant weight and their initial dry weights were recorded using an analytical balance (±0.01 g). The specimens were then horizontally buried in the soil and allowed for a total period of 90 days under actual conditions. At each of the specified periods (15, 30, 45, 60, 75, and 90 days), samples were carefully removed from the soil, gently cleaned with water to wash out adhered soil particles, and dried at 70 °C until a constant weight was achieved. The final dry weights were then measured, and the percentage weight loss was calculated using the following equation: Total weight loss (%) = [(Initial weight at day 0 − Final weight at each sampling day)/Initial weight at day 0] × 100 [24].

#### 2.6.2. Phytotoxicity Test

The phytotoxicity test was conducted under greenhouse conditions to assess the possible impacts of MBCP on crop development. After mechanical testing, each MBCP sample was ground into fine particles and sieved through a 1 mm mesh to obtain a consistent powder form. The obtained material was then mixed with sterilized commercial potting soil at a ratio of 10 g/kg [25]. Four crop species were selected as test plants, including chili pepper, Chinese cabbage, holy basil, and tomato. Prior to growing, the seeds were surface-sterilized by immersion in a 2% sodium hypochlorite solution with a few drops of Tween-20 for 2 min, followed by rinsing twice with sterile distilled water to remove any residual contaminants.

For each treatment, plastic seedling trays (30 cm × 60 cm × 3.5 cm) were filled loosely with 1 kg of prepared soil mixture. A total of 100 seeds (divided into four sections of 25 seeds each) were evenly distributed on the soil surface, lightly covered with a thin layer of sterile silica sand, and the trays were then covered with transparent plastic film to minimize moisture loss. After 7 days, the plastic film was removed once the developing seedlings contacted its surface. The seedlings were subsequently grown under controlled greenhouse conditions for 21 days, maintained at 60–70% relative humidity. At the end of the cultivation period, seedling performance was assessed in terms of germination percentage, seedling height, and root length [26,27].

### 2.7. Drop Test of Loaded Containers Under Free-Fall Conditions

The packaging performance of the developed cushioning materials was evaluated by simulating the mechanical stresses and handling impacts typically encountered during storage, handling, and transportation. All drop tests were conducted under laboratory conditions using a modified procedure based on ASTM D5276 (Standard test method for drop test of loaded containers by free fall) to assess the protective capability of the developed packaging systems (Figure 1).

The dimensional and geometric characteristics of the six packaging treatments evaluated in this study are summarized in Table 1. All packaging forms exhibited a cylindrical geometry and provided 100% coverage of the bottle circumference. The measured parameters included packaging thickness, inner and outer diameters, height, depth, internal volume, coverage area, and the gap between the bottle and the inner packaging wall. These parameters were used to characterize the dimensional compatibility and suitability of each packaging form with the test bottle.

The drop height was determined according to the package weight. Since all test packages weighed less than 10 kg, a drop height of 0.76 m was applied. Each packaging treatment was subjected to 10 free-fall drops, comprising one corner drop, three edge drops, and six face drops. All tests were performed manually and recorded on video for subsequent evaluation (Figure 2) [28]. Three replicate packages were tested for each of the six cushioning treatments during the preliminary performance assessment. The damage evaluation and protection performance data were recorded according to the adapted drop-test procedure reported by Innopack [29]. As this was an initial assessment, damage was evaluated by subjective visual inspection and recorded using a simple categorical scoring approach: No = no visible damage; Yes = visible damage; and − = the damage category was not applicable or was not observed/recorded. The observations from all drop events were subsequently compiled to compare the protective performance of the different cushioning treatments.

### 2.8. Statistical Analysis and Cost Comparison of the Developed Specimens

The experimental data were analyzed using one-way analysis of variance (ANOVA) with SPSS for Windows (version 17.0; SPSS Inc., Chicago, IL, USA). When significant differences were observed (*p* < 0.05), Duncan’s multiple range test was applied to determine differences among treatment means. In addition, the overall performance of the MBCP was evaluated in terms of both material properties and production cost, and subsequently compared with those of conventional materials to assess their potential for practical applications.

## 3. Results and Discussions

### 3.1. Physical Properties of Developed MBCP

The primary physical properties of MBCP produced from different lignocellulosic biomass residues combined with *L. sajor-caju* are summarized in Table 2. Significant differences were observed among substrate types in terms of volumetric shrinkage, dried weight, and dried density, demonstrating that biomass type strongly influences the structural development and final physical characteristics of the composites.

Volumetric shrinkage ranged from 5.23% to 15.29%, with the highest shrinkage recorded in corn husk-based MBCP (15.29%), followed by rice straw (13.44%), whereas sawdust (5.23%) and sugarcane leaf (5.55%) composites exhibited significantly lower shrinkage. The greater dimensional reduction observed in corn husk and rice straw composites is likely associated with their relatively loose fibrous structures, low density, and higher internal porosity, which facilitate moisture loss during drying and result in greater structural contraction [30,31,32]. In contrast, the denser particle packing and higher lignin content of sawdust may provide greater dimensional stability by reducing significant shrinkage during moisture evaporation [32,33]. Similar relationships between substrate composition, drying behavior, and dimensional stability have been reported in previous studies on MBCs, where lignocellulosic particle characteristics significantly influenced composite morphology and structural integrity [11,13,20].

The dried weight of the developed MBCP differed significantly among substrate treatments. Sawdust-based MBCP exhibited the highest dried weight (38.74 g), whereas corn husk and rice straw composites showed comparable values of 29.26 and 28.43 g, respectively. The lowest dried weight was observed in sugarcane leaf-based MBCP (26.32 g). Consequently, sawdust-based MBCP achieved the highest dried density (314.79 kg/m^3^), followed by corn husk (243.18 kg/m^3^) and sugarcane leaf (229.80 kg/m^3^), while rice straw produced the lightest composite (216.49 kg/m^3^). These differences demonstrate the intrinsic physicochemical characteristics of the biomass residues, including particle size distribution, cellulose–hemicellulose–lignin composition, and the ability of fungal hyphae to colonize and interconnect substrate particles [9,34]. Sawdust generally forms compact composite matrices because its fine particles create a larger contact area for hyphal adhesion, resulting in greater material consolidation and density. Conversely, the elongated fibers of rice straw generate a more open internal structure with larger interconnected voids, thereby reducing the overall density [11].

From an application perspective, the observed variation in density provides opportunities to tailor MBCP for different packaging requirements. Higher-density composites, such as those produced from sawdust, are expected to exhibit greater mechanical strength and load-bearing capacity, making them suitable for rigid protective packaging. In contrast, lower-density composites derived from corn husk, rice straw, and sugarcane leaves may offer improved cushioning performance due to their more porous structures while simultaneously reducing material consumption and product weight. Importantly, all agricultural residues successfully supported extensive mycelial growth and composite formation, demonstrating their potential as renewable feedstocks for sustainable packaging materials. These findings highlight that careful selection of biomass residues represents an effective strategy for engineering the physical properties of MBCs, thereby expanding their potential to replace petroleum-based plastic and foam packaging within a circular bioeconomy.

The water absorption and volumetric swelling behavior of the developed MBCP varied significantly depending on the lignocellulosic substrate used (Figure 3). Water absorption increased markedly with immersion time for all treatments, indicating the hydrophilic nature of both the lignocellulosic fibers and the *L. sajor-caju* mycelial network. After 2 h of immersion, the rice straw-based MBCP (T2) exhibited the highest water absorption (approximately 165%), followed by sugarcane leaf (T4; ~110%), corn husk (T1; ~100%), and sawdust (T3; ~82%). A similar trend was observed after 24 h, with T2 showing the greatest water uptake (approximately 210%), whereas T3 maintained the lowest value (approximately 125%). Corn husk- and sugarcane leaf-based MBCP displayed intermediate absorption levels of approximately 162% and 150%, respectively. These results demonstrate that the substrate composition significantly influenced the moisture resistance of the composites.

The volumetric swelling results followed a similar pattern. Rice straw-based MBCP (T2) exhibited the greatest dimensional expansion (approximately 8.6%), while sawdust-based MBCP (T3) showed the lowest swelling (approximately 4.5%). Corn husk (T1) and sugarcane leaf (T4) composites displayed intermediate swelling values of approximately 6.0% and 5.9%, respectively, with no significant difference between these treatments. The relatively low swelling observed in T3 indicates superior dimensional stability under wet conditions.

The differences in water absorption and swelling can be attributed to variations in substrate microstructure, chemical composition, and the degree of mycelial binding [9,35]. Rice straw possesses a highly porous structure with abundant capillary spaces that facilitate rapid water penetration, leading to greater moisture uptake and dimensional expansion. This observation is consistent with previous studies reporting that low-density MBCs generally exhibit higher porosity, enabling easier water infiltration and consequently greater water absorption and swelling [7,33]. In contrast, sawdust forms a denser and more compact composite due to its finer particle size and stronger interfacial bonding with the mycelial network, thereby restricting water diffusion and limiting swelling [10,12]. The intermediate behavior of corn husk and sugarcane leaf composites suggests a proportionate pore structure that provides moderate moisture resistance while maintaining sufficient porosity for lightweight cushioning applications.

Overall, the results indicate that although all MBCP readily absorbed water because of their biodegradable lignocellulosic nature, substantial differences existed in their moisture stability. Sawdust-based MBCP exhibited the best resistance to water absorption and dimensional change, making it particularly suitable for packaging applications where structural stability under humid conditions is required. Conversely, the higher moisture uptake of rice straw-based composites suggests that additional surface treatments or hydrophobic coatings may be necessary to improve their durability for practical packaging applications. These findings highlight the importance of substrate selection in optimizing the balance between sustainability, biodegradability, and functional performance of mycelium-based packaging materials. Nevertheless, the high-water absorption capacity of certain MBCP may also provide a functional advantage in specialized applications, such as absorbent cushioning materials or protective packaging for liquid-based substances, where rapid absorption of leaked liquids during transportation can help minimize harm and improve handling safety [13].

### 3.2. External and Internal Surface Microstructures of Developed MBCP

Scanning electron microscopy revealed distinct differences in the external and internal surface architectures of the developed MBCP, reflecting the influence of lignocellulosic substrate characteristics on mycelial colonization and composite development (Figure 4). Overall, all matrices exhibited a multidimensional network of intertwined *L. sajor-caju* hyphae that interconnected adjacent biomass particles, confirming successful substrate colonization and the formation of a continuous natural binding matrix. However, the density, uniformity, and pore distribution of the mycelial network varied considerably among substrate types.

The corn husk-based MBCP (T1) displayed a well-developed fibrous network with numerous interconnected pores on both the external and internal surfaces. The hyphae were evenly distributed, producing a porous yet cohesive structure that is advantageous for dissipating impact energy while maintaining structural integrity. In contrast, the rice straw-based MBCP (T2) exhibited a comparatively enormous surface area with biomass particles partially covered by a thinner mycelial layer. The reduced pore connectivity and incomplete hyphal bonding suggest weaker substrate integration, which may contribute to the lower mechanical stability and greater deformation observed during shock loading.

The sawdust-based MBCP (T3) presented a dense and highly interconnected mycelial matrix with extensive hyphal branching throughout both surfaces. The continuous hyphal network effectively covered the wood-based particles, producing a compact composite with fewer structural discontinuities. Such morphology is consistent with the enhanced compressive resistance of sawdust-derived composites, although the increased rigidity may reduce their ability to absorb impact energy through elastic deformation. Conversely, the sugarcane leaf-based MBCP (T4) showed a relatively loose but uniformly distributed network of elongated hyphae that formed an open fibrous architecture with abundant interconnected voids. This porous structure is likely to promote efficient stress redistribution and energy dissipation during compression and drop impacts.

These observations demonstrate that substrate morphology strongly governs mycelial growth behavior and the resulting microstructure of MBCP. A well-connected hyphal network serves as a natural adhesive that binds lignocellulosic fibers into a combined composite [36], while the balance between pore size and network density determines the trade-off between stiffness and cushioning performance [37]. The superior microstructural characteristics observed in the corn husk and sugarcane leaf composites agree with their favorable impact resistance, whereas the denser sawdust composite corresponds to greater structural rigidity. Overall, the SEM analysis confirms that tailoring substrate composition provides an effective strategy for engineering mycelium-based packaging materials with optimized internal architectures and functional performance, offering a sustainable alternative to conventional petroleum-based cushioning materials.

### 3.3. Mechanical Properties of Developed MBCP

The ultimate compressive strength of the developed MBCP differed significantly among the lignocellulosic substrates used (Table 3), demonstrating that substrate selection plays a critical role in determining the load-bearing capacity of the composites. The highest compressive strength was obtained from MBCP produced using sawdust (T3), reaching 0.91 MPa, followed by sugarcane leaf-based composites (T4, 0.68 MPa). In contrast, corn husk (T1) and rice straw (T2) produced significantly lower compressive strengths of 0.43 MPa and 0.29 MPa, respectively. These results indicate that the mechanical performance of MBCP is highly dependent on the physicochemical characteristics and structural composition of the biomass substrate.

The superior compressive strength of sawdust-based MBCP can be attributed to its relatively high lignin content, finer particle size, and greater packing density, which facilitate stronger interfacial bonding between fungal hyphae and substrate particles [10,38]. A denser and more continuous mycelial network effectively distributes compressive stress throughout the composite matrix, thereby enhancing structural integrity and resistance to deformation [8,39]. Sugarcane leaf-based MBCP also exhibited good mechanical performance, likely due to its fibrous architecture, which promotes effective mycelial colonization and reinforcement of the internal structure [40,41].

Conversely, the lower compressive strengths observed in corn husk- and rice straw-based composites are likely associated with their coarser particle morphology, lower packing efficiency, and the formation of larger internal voids. These structural characteristics reduce the continuity of the mycelial matrix and limit efficient stress transfer under compressive loading [34,42,43]. Similar relationships between substrate morphology, mycelial network density, and mechanical performance have been reported in previous studies, where denser composites consistently exhibited superior compressive properties [44,45,46]. Overall, these findings demonstrate that the selection of appropriate lignocellulosic residues plays a critical role in optimizing the compressive performance of MBCP for protective packaging applications. Among the substrates evaluated, sawdust-based composites exhibited the highest compressive strength, highlighting their strong potential as sustainable alternatives to conventional petroleum-derived foam packaging, particularly for protecting high-value and fragile products during handling and transportation. Moreover, the developed MBCP mostly exhibited compressive strengths comparable to or exceeding those of several widely used commercial foam packaging materials, including expanded polyethylene (0.05–0.16 MPa), expanded polystyrene (0.04–0.46 MPa), expanded polypropylene (0.30–2.50 MPa), and rigid polyurethane (0.08–0.24 MPa) [47,48,49,50]. These results highlight the potential of the developed MBCP to serve as environmentally sustainable packaging materials that combine adequate mechanical protection with biodegradability and reduced environmental impact.

### 3.4. Biodegradability and Phytotoxicity of Developed MBCP

#### 3.4.1. Soil Burial Degradation

The soil burial test demonstrated that all developed MBCP underwent progressive biodegradation throughout the 90-day incubation period, although the degradation rate varied significantly among substrate formulations (Figure 5). Cumulative weight loss increased continuously with burial duration in all treatments (Figure 5A), indicating the gradual decomposition of the fungal–lignocellulosic matrix under natural soil conditions. The highest biodegradation was observed in T1 and T4, reaching approximately 64.2% and 61.8% cumulative weight loss after 90 days, respectively, followed by T2 (52.1%), whereas T3 exhibited the greatest resistance to degradation, with only 30.3% weight loss. Significant differences among treatments were detected at each sampling period, indicating that substrate formulation substantially influenced the degradation behavior of the composites. Notably, T1 and T4 exceeded 60% mass loss within 90 days, approaching the commonly referenced biodegradation criterion of ≥60% degradation within 3–6 months for biodegradable materials [51]. These findings highlight the potential of the developed MBCP, particularly T1 and T4, for environmentally compatible applications where end-of-life biological degradation is desirable.

The degradation rates further revealed that the greatest weight loss occurred during the first 15 days of burial (Figure 5B), where T1 and T4 exhibited rapid decomposition, with weight losses exceeding 23–25%, significantly higher than T2 and T3. Thereafter, the degradation rate gradually declined in all treatments, with weight loss decreasing progressively between 30 and 90 days. By the final interval (60–90 days), only limited additional mass loss was observed, indicating that the readily degradable constituents had largely decomposed, while the remaining material consisted predominantly of more recalcitrant lignocellulosic fractions and residual fungal biomass.

The slower degradation observed in T3 suggests the formation of a more compact and structurally stable composite matrix that limited moisture penetration and microbial accessibility, thereby enhancing durability under soil conditions. Previous studies have reported that increased compaction and higher mycelial density may prolong biodegradation by restricting microbial colonization and slowing the disintegration of the composite matrix [52]. In contrast, the faster degradation of T1 and T4 may be attributed to their relatively polymeric structure and greater accessibility of biodegradable lignocellulosic constituents, facilitating enzymatic breakdown and microbial attack [53,54]. These findings are consistent with previous studies reporting that the biodegradation behavior of MBCs is governed by substrate composition, porosity, density, degree of mycelial binding, and microbial activity [12,53,55]. Collectively, the results demonstrate that substrate compositions can effectively regulate the trade-off between structural durability during use and rapid biodegradation after disposal, highlighting the potential of the developed MBCP as environmentally sustainable alternatives to petroleum-based packaging materials within a circular bioeconomy framework.

#### 3.4.2. Phytotoxicity

The phytotoxicity assessment demonstrated that soil amended with MBCP powder had no adverse effects on seed germination or early seedling development of the tested plant species (Figure 6). Across all treatments, seed germination percentages remained consistently high, exceeding 90% for chili pepper, Chinese cabbage, holy basil, and tomato, with no significant differences between the control and MBCP samples. These results indicate the absence of phytotoxic compounds released from the developed composites, confirming their biological compatibility and environmental safety. Although germination was unaffected, significant differences were observed in seedling growth responses, particularly radicle elongation. In chili pepper, all MBCP samples significantly promoted root development compared with the control, with the rice straw-based MBCP (T2) producing the longest radicles, followed by T3, T4, and T1. Stem length also increased slightly in all treatments, although the differences were less pronounced. A similar growth-promoting effect was observed in Chinese cabbage, where T3 and T4 produced the greatest radicle elongation, while all MBCP treatments significantly enhanced stem length relative to the control. These findings suggest that soluble compounds obtained from the composites could help promote early root establishment in rapidly growing vegetable species.

In holy basil, stem length was significantly improved in all treatments compared with the control, whereas radicle length showed only moderate increases, with T1 producing the highest value. Tomato seedlings exhibited the least variation among treatments, although stem growth was consistently greater than in the control, while radicle length remained statistically unchanged. The relatively stable response of tomato indicates that the samples neither inhibited nor overstimulated seedling development.

The positive effects observed in several plant species may be attributed to the gradual release of water-soluble nutrients and beneficial components generated during fungal colonization of the lignocellulosic substrates. The degradation of cellulose and hemicellulose by *L. sajor-caju* likely produces low-molecular-weight organic compounds and mineral nutrients that can enhance early root growth without introducing toxic residues [56,57,58]. Furthermore, the extensive mycelial biotransformation process may reduce or eliminate naturally occurring phytotoxic compounds present in raw agricultural residues [59], thereby improving the biological quality of the final composite. Overall, the phytotoxicity results confirm that all developed MBCP samples are environmentally benign and exhibit excellent biocompatibility with plants. Their ability to maintain high seed germination while promoting seedling growth in several species highlights their potential as sustainable packaging materials that can be repurposed as biodegradable planters and safely incorporated into the soil after use. However, these phytotoxicity assays primarily demonstrate compatibility with plants and do not directly establish human-health safety during handling or unpacking. In particular, potential exposure to surface-level mycelial particles, residual low-molecular-weight metabolites, or fine particles generated by mechanical abrasion of the dried composites was not evaluated in the present study. Therefore, further investigations, including allergenicity, general cytotoxicity, and airborne-particle or dust-exposure assessments, are warranted before the materials can be considered fully validated for direct human-contact applications. Nevertheless, the favorable plant-compatibility and biodegradable end-of-life characteristics observed in this study further support the potential advantages of mycelium-based composites over conventional petroleum-derived plastic and foam packaging, particularly within a circular bioeconomy framework.

### 3.5. Free-Fall Drop Test

The free-fall drop test was conducted to evaluate the impact resistance and protective performance of the developed MBCP in comparison with conventional packaging materials, namely air bubble wrap and EPE foam (Table 4). All packaging systems were subjected to a drop height of 0.76 m while protecting a glass bottle containing 80 mL of water. Overall, none of the tested samples resulted in leakage or cracking of the glass bottle, indicating that all cushioning materials provided a basic level of impact protection under the applied test conditions.

The conventional air bubble wrap (T5) and EPE foam (T6) exhibited the lowest empty weights, ranging from 5.15 to 9.46 g, reflecting their lightweight nature. In contrast, the developed MBCP possessed significantly greater masses (51.65–79.25 g), with the sawdust-based specimen (T3) being the heaviest and the sugarcane leaf-based specimen (T4) the lightest. The higher weight and density of the MBCP can be attributed to their lignocellulosic matrix and dense mycelial network, which contribute to structural stiffness and energy transfer during impact [28].

Among the developed materials, the corn husk-based MBCP (T1) demonstrated the most promising cushioning performance. After the impact test, no lid failure, deformation, scratches, or cracks after impact were observed on the protected bottle, indicating effective protection against mechanical damage. A minor scratch was observed on the surface of the MBCP cushioning material itself, but this superficial damage did not compromise the integrity or protective function of the packaging. Similarly, the sugarcane leaf-based MBCP (T4) prevented bottle damage and lid failure, although slight deformation of the cushioning structure was observed. In contrast, the rice straw (T2) and sawdust (T3) specimens showed more structural loss, including deformation and surface damage, while the bottle lid also failed in these treatments. These observations indicate that the cushioning efficiency of MBCP is strongly influenced by substrate composition and the resulting internal architecture formed by mycelial colonization.

Interestingly, although air bubble wrap and EPE foam effectively prevented bottle breakage, lid failure was still observed, implying limited restraint against sudden impact-induced movement [60,61,62]. Conversely, the corn husk and sugarcane leaf MBCP provided improved directional protection and impact distribution, thereby reducing secondary damage to the packaged product (Figure 7). The superior performance of these biomass-derived composites may be associated with their interconnected porous structure, which enables progressive snugness and enhanced energy absorption during loading [28].

These findings demonstrate that properly engineered MBCP can achieve impact resistance comparable to, or in some aspects better than, conventional plastic-based cushioning materials. In particular, the corn husk-based specimen exhibited excellent protective performance while utilizing an abundant agricultural residue, highlighting its potential as a sustainable and biodegradable alternative for protective packaging applications. The results further support the growing feasibility of replacing petroleum-derived cushioning materials with MBCs, contributing to recurrent green economy initiatives and reducing dependence on non-renewable packaging resources.

### 3.6. Comparative Evaluation and Recommendations

The comparative assessment demonstrated clear differences between conventional petroleum-derived packaging materials and the developed MBCP (Table 5). Air bubble wrap and plastic foam offer advantages in lightweight design, minimal material usage, and versatility for generic packaging applications. However, these materials are derived from non-renewable petroleum resources and exhibit limited biodegradability, contributing to persistent plastic waste. In contrast, the developed MBCP fulfills several key principles of sustainable packaging by being biodegradable, recyclable, and manufactured from renewable lignocellulosic waste materials. Furthermore, MBCP can be fabricated into custom-molded protective packaging, including trays, corner guards, and inserts, providing effective cushioning for fragile products while reducing environmental impacts. However, the economic feasibility of MBCP remains a challenge due to its 21-day incubation period, energy requirements for maintaining 30 °C, and labor-intensive molding process, despite the potential for low-cost biomass feedstocks. Therefore, further optimization of substrate formulation, moisture resistance, incubation time, molding, and process automation is needed to improve production efficiency and scalability. Overall, MBCP shows strong potential as an eco-friendly packaging alternative, particularly for applications where biodegradability, waste valorization, and custom molding are prioritized over ultra-high production speed and minimum material use. These advances could facilitate its integration into sustainable and circular-based packaging solutions.

From an economic perspective, the finished-product cost of the developed MBCP was approximately 14.50 THB (USD 0.4391) per package, compared with 0.17 THB (USD 0.0052) for air bubble wrap and 21.40 THB (USD 0.6420) for plastic foam under the production conditions and local market parameters considered in this study (Table 5). These values indicate that the economic position of MBCP ranges midway between the two conventional materials. Although the MBCP cost was substantially higher than that of air bubble wrap, it was lower than that of the evaluated plastic foam product. However, this comparison should be interpreted cautiously because the costs were based on the specific production conditions and local product prices considered in the present study and may differ across regions, production scales, and commercial sectors.

An additional economic consideration is the substantially greater mass of MBCP. The developed MBCP specimens weighed approximately 5–10 times more than conventional packaging materials, which can increase transportation and logistics costs when packaging is transported over long distances. Therefore, the economic evaluation should not be based only on the manufacturing cost per package. The higher mass may increase freight requirements, handling costs, and associated transportation-related environmental impacts. This factor is particularly important for applications involving large distribution distances or high-volume shipments. Consequently, reducing the density and material mass of MBCP while maintaining suitable cushioning and mechanical protection should be a priority for future development.

Although further improvements regarding material optimization, moisture resistance, production efficiency, and manufacturing scalability are still required, the environmental safety of MBCP should also be carefully evaluated, particularly with respect to the potential bioaccumulation of heavy metals by fungi from contaminated growth substrates. Therefore, future studies should include chemical purity and trace heavy-metal analyses of both the mycelium and final composite, using techniques such as SEM–EDS for preliminary screening and ICP-MS or AAS for quantitative assessment. In addition, research should focus on optimizing composite combinations, enhancing durability, and developing cost-effective large-scale production systems to facilitate broader practical acceptance and support the transition toward a circular-based green economy.

## 4. Conclusions

This study demonstrates that MBCP produced from *L. sajor-caju* and abundant biomass residues represents a promising sustainable alternative to conventional petroleum-based plastic and foam packaging. The physical, microstructural, mechanical, biodegradation, phytotoxicity, and cushioning performances of the composites were strongly influenced by substrate selection, enabling the development of packaging materials with tailored functional properties. Sawdust-based MBCP exhibited the highest density, compressive strength, and durability to moisture, making it suitable for applications requiring high structural stability. In contrast, corn husk- and sugarcane leaf-based MBCP developed interconnected porous architectures that provided excellent impact absorption and protective performance during free-fall testing, comparable to or surpassing conventional cushioning materials. All developed MBCP was biodegradable under soil burial conditions and showed no phytotoxic effects, while several treatments promoted early seedling growth, highlighting their environmental compatibility and potential for safe end-of-life disposal or recycling as biodegradable planters. These findings demonstrate that biomass residues can be successfully valorized into high-value packaging materials through fungal biotransformation, simultaneously reducing agricultural waste and dependence on non-renewable resources. Although improvements in moisture resistance, manufacturing efficiency, and large-scale production remain necessary, the developed MBCP provides a strong foundation for next-generation sustainable packaging. All things considered, this work advances the development of renewable, biodegradable, and high-performance MBCP materials that contribute to reducing the environmental footprint of the packaging industry.

## Figures and Tables

**Figure 1:**
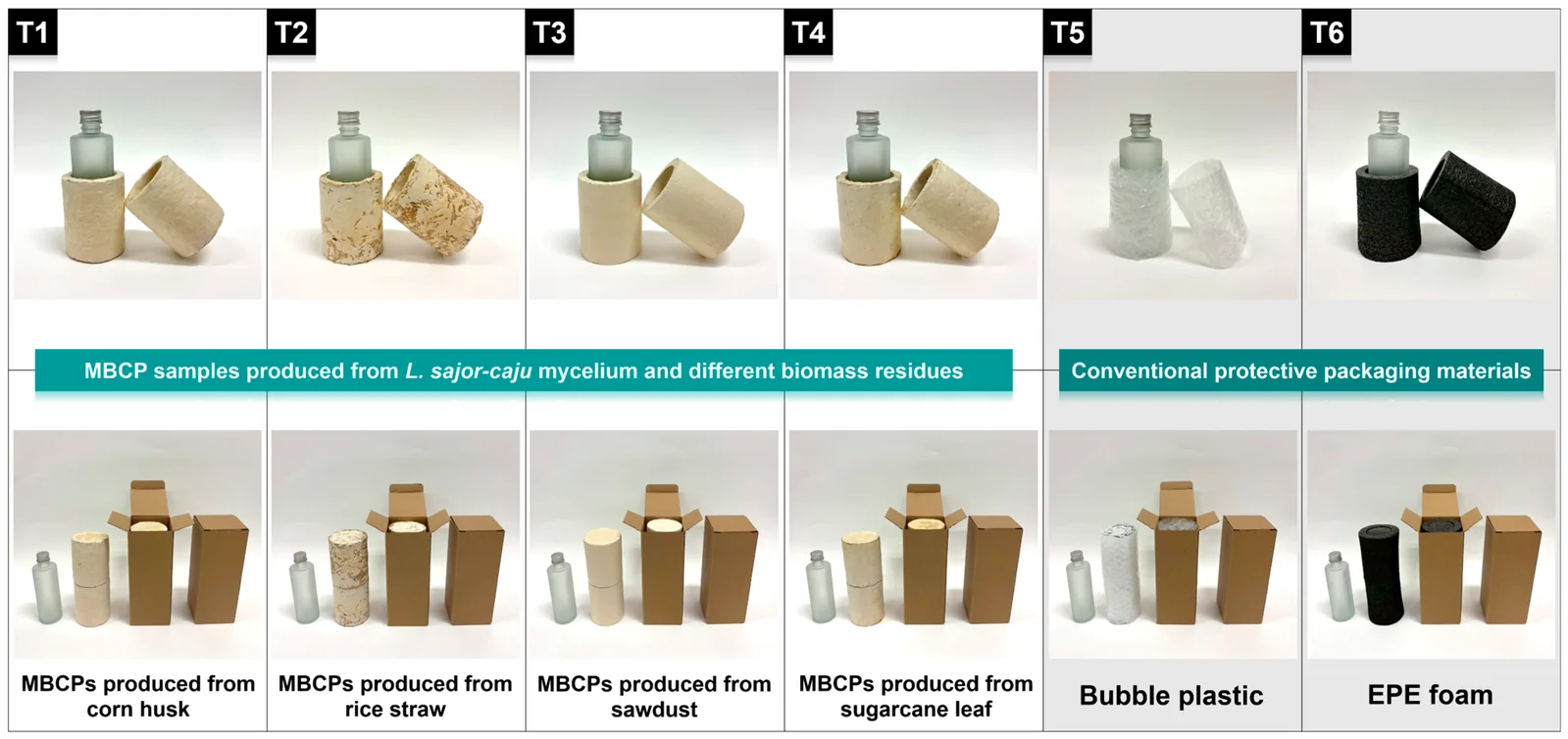
Representative conventional protective packaging materials and the developed MBCP fabricated from *L. sajor-caju* mycelium cultivated on different lignocellulosic biomass residues. (T1) MBCP produced from corn husk; (T2) MBCP produced from rice straw; (T3) MBCP produced from sawdust; (T4) MBCP produced from sugarcane leaf; (T5) air bubble wrap; and (T6) EPE foam.

**Figure 2:**
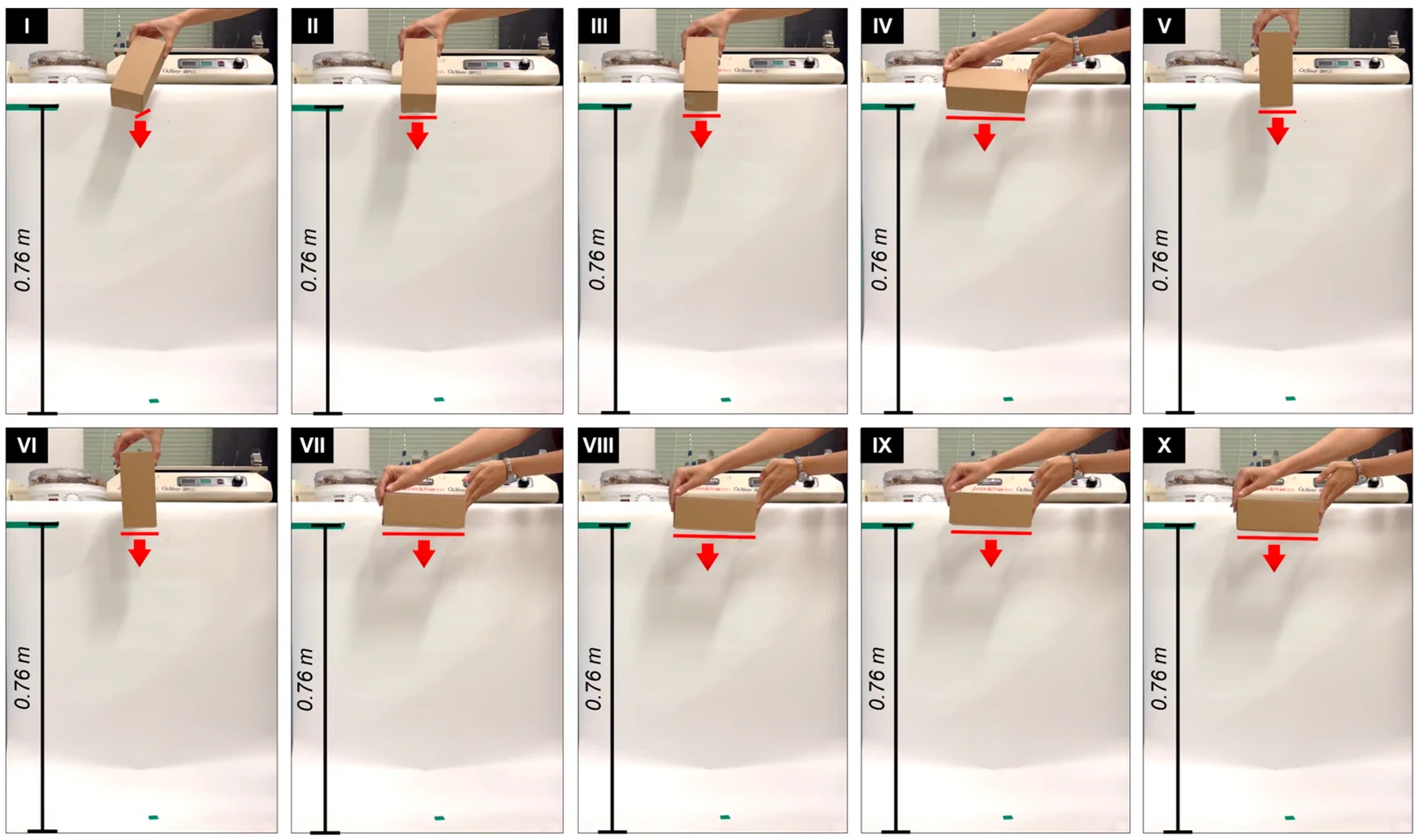
Schematic illustration of the 10 free-fall drop positions, comprising one corner drop, three edge drops, and six face drops: I = most fragile corner; II = shortest edge radiating from the drop corner; III = medium edge radiating from the drop corner; IV = longest edge radiating from the drop corner; V = one of the smallest faces; VI = opposite smallest face; VII = one of the medium faces; VIII = opposite medium face; IX = one of the largest faces; and X = opposite largest face.

**Figure 3:**
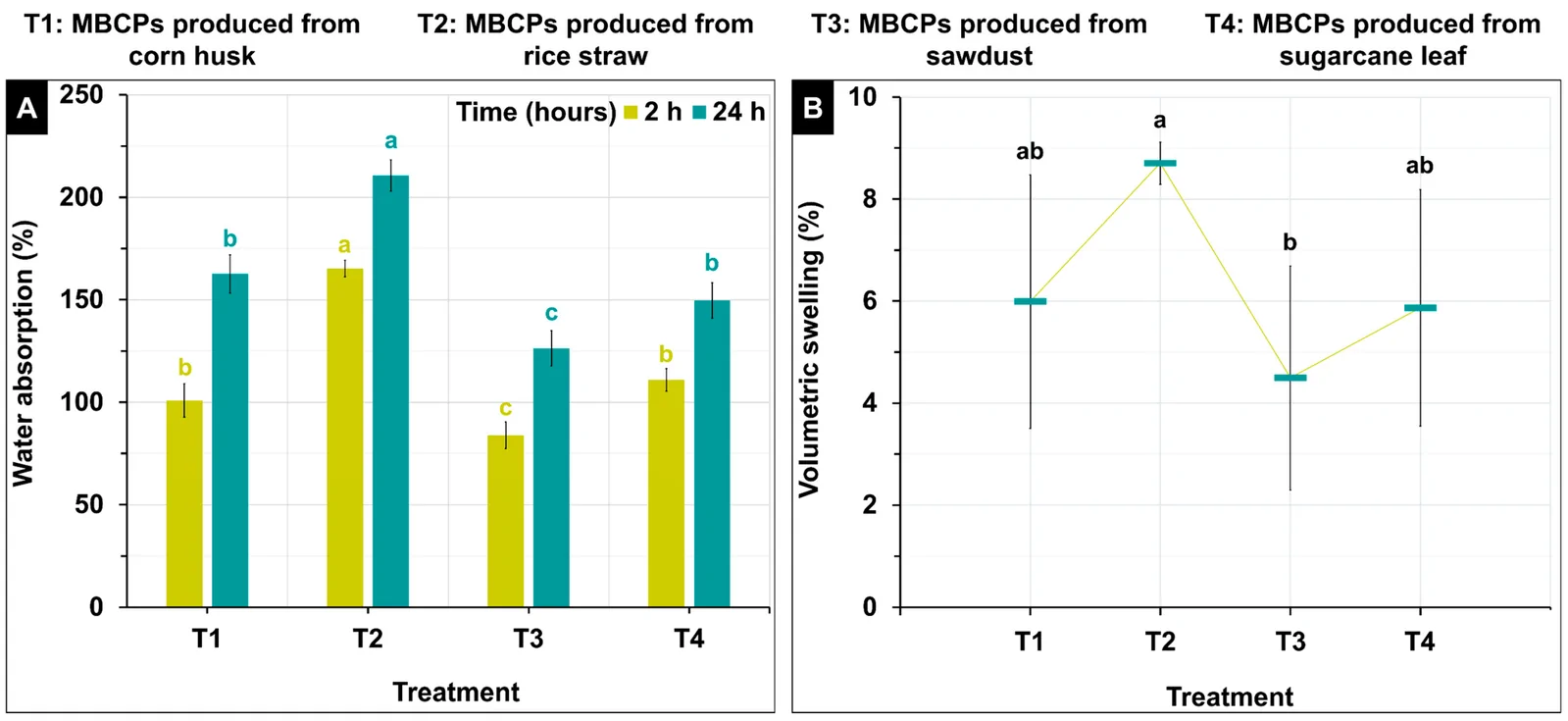
Water absorption (**A**) and volumetric swelling (**B**) of MBCP produced from different lignocellulosic biomass residues combined with *Lentinus sajor-caju* mycelium. Treatments consisted of T1: corn husk-based MBCP, T2: rice straw-based MBCP, T3: sawdust-based MBCP, and T4: sugarcane leaf-based MBCP. Data are presented as mean ± standard deviation. Different lowercase letters indicate significant differences among treatments according to Duncan’s multiple range test (*p* < 0.05).

**Figure 4:**
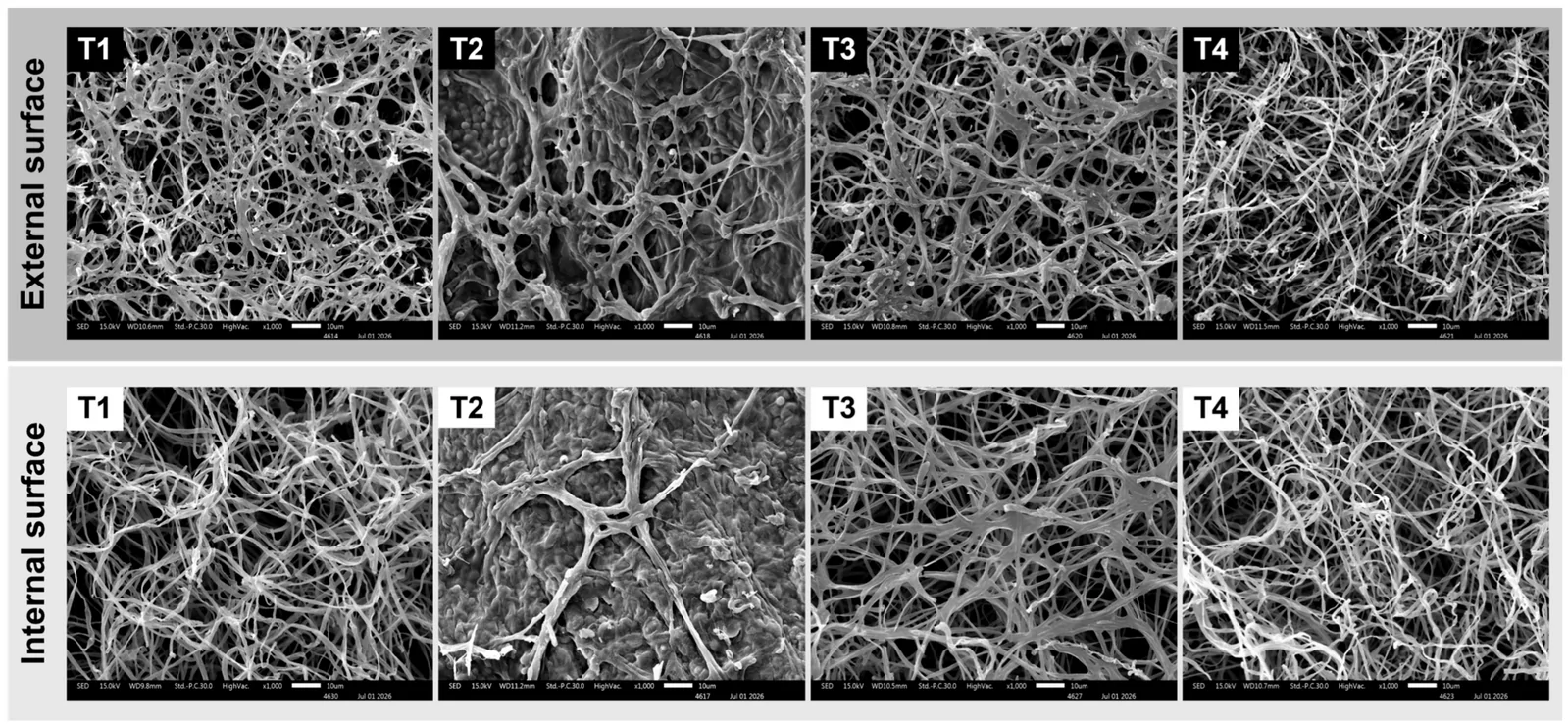
Scanning electron microscopy images of the external and internal surface microstructures of the developed MBCP. T1: Corn husk-based MBCP; T2: Rice straw-based MBCP; T3: Sawdust-based MBCP; and T4: Sugarcane leaf-based MBCP.

**Figure 5:**
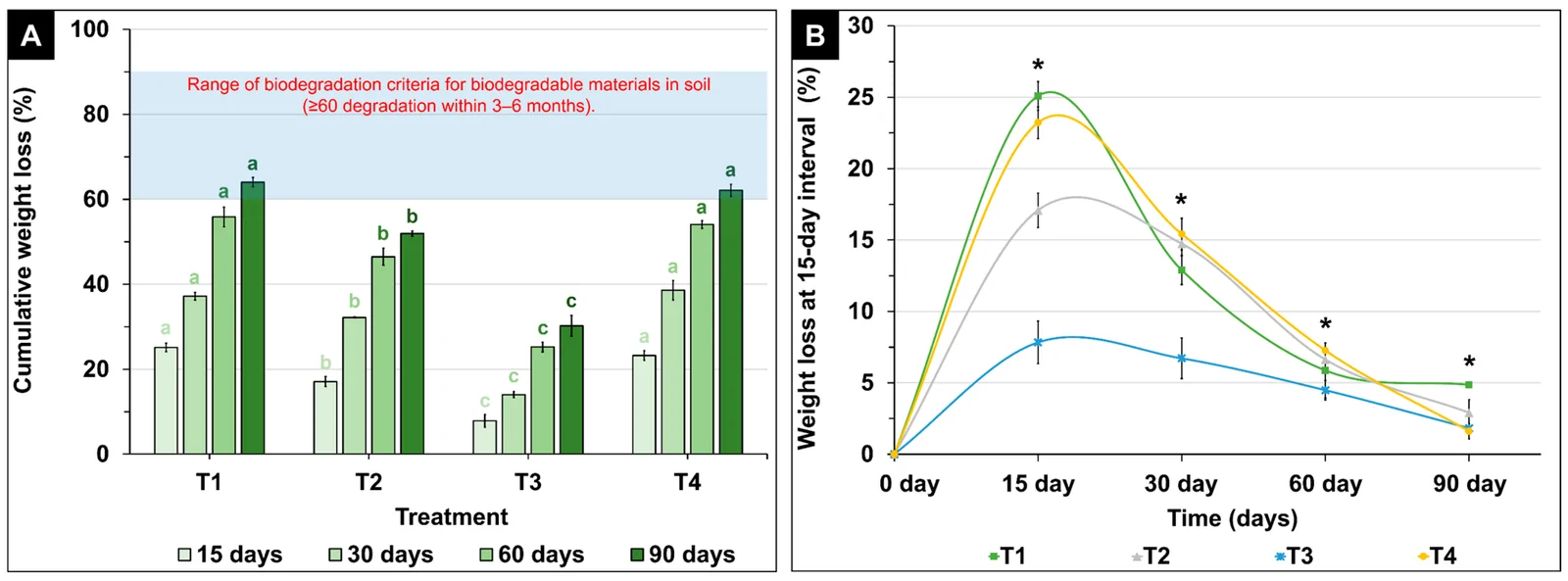
Cumulative weight loss (**A**) and weight loss during each 30-day interval (**B**) of the developed MBCP during the soil burial biodegradation test in commercial potting soil. Data are presented as the mean ± standard deviation. Different letters in (**A**) and an asterisk (*) in (**B**) denote significant differences among treatments at each sampling time according to Duncan’s multiple range test (*p* < 0.05).

**Figure 6:**
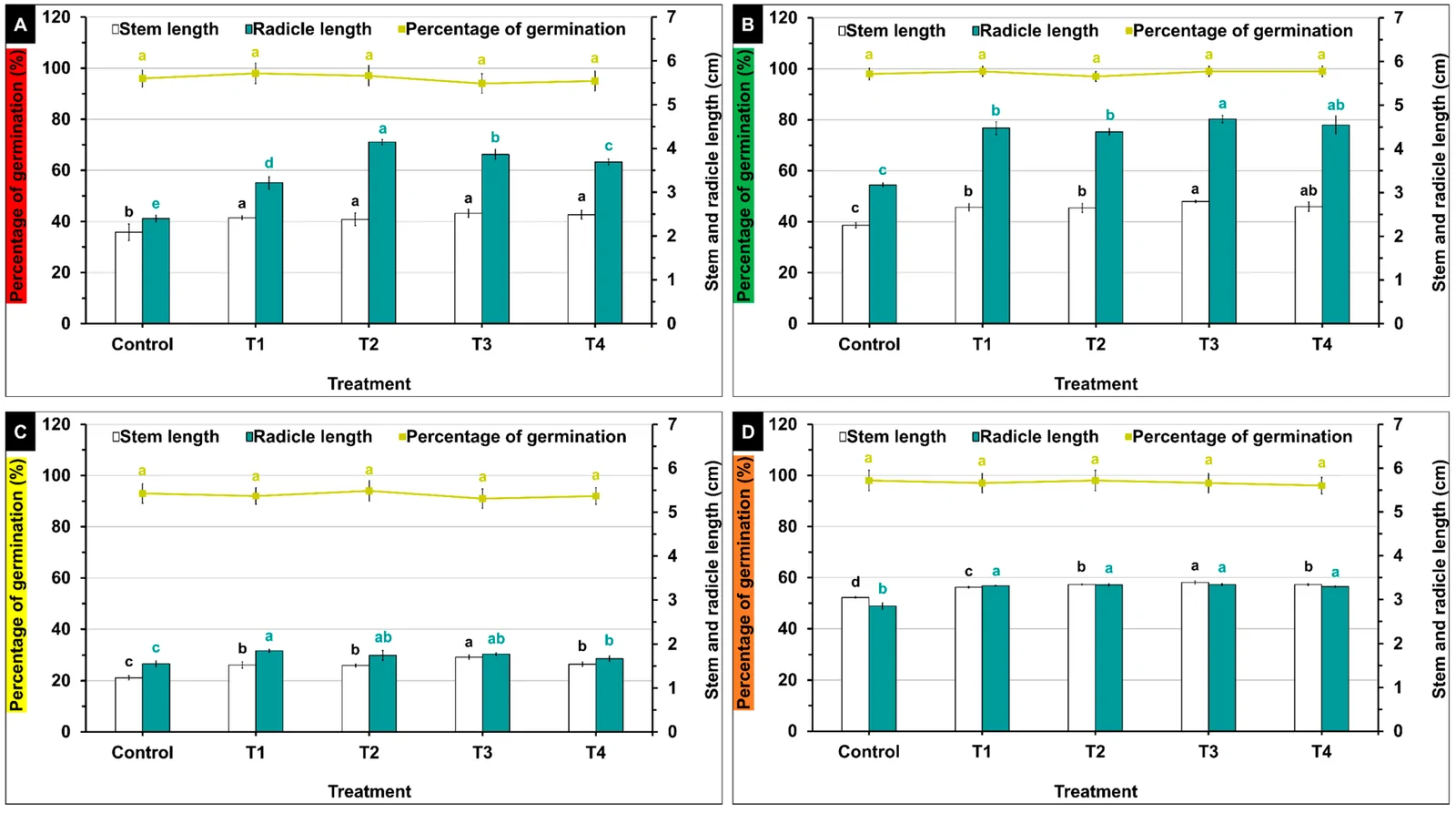
Effects of MBCP on seed germination (%), stem length, and radicle length of four plant species used in the phytotoxicity assay: (**A**) chili pepper, (**B**) Chinese cabbage, (**C**) holy basil, and (**D**) tomato. Values are expressed as mean ± standard deviation. Different letters within the same parameter for each plant species indicate significant differences according to Duncan’s multiple range test (*p* < 0.05).

**Figure 7:**
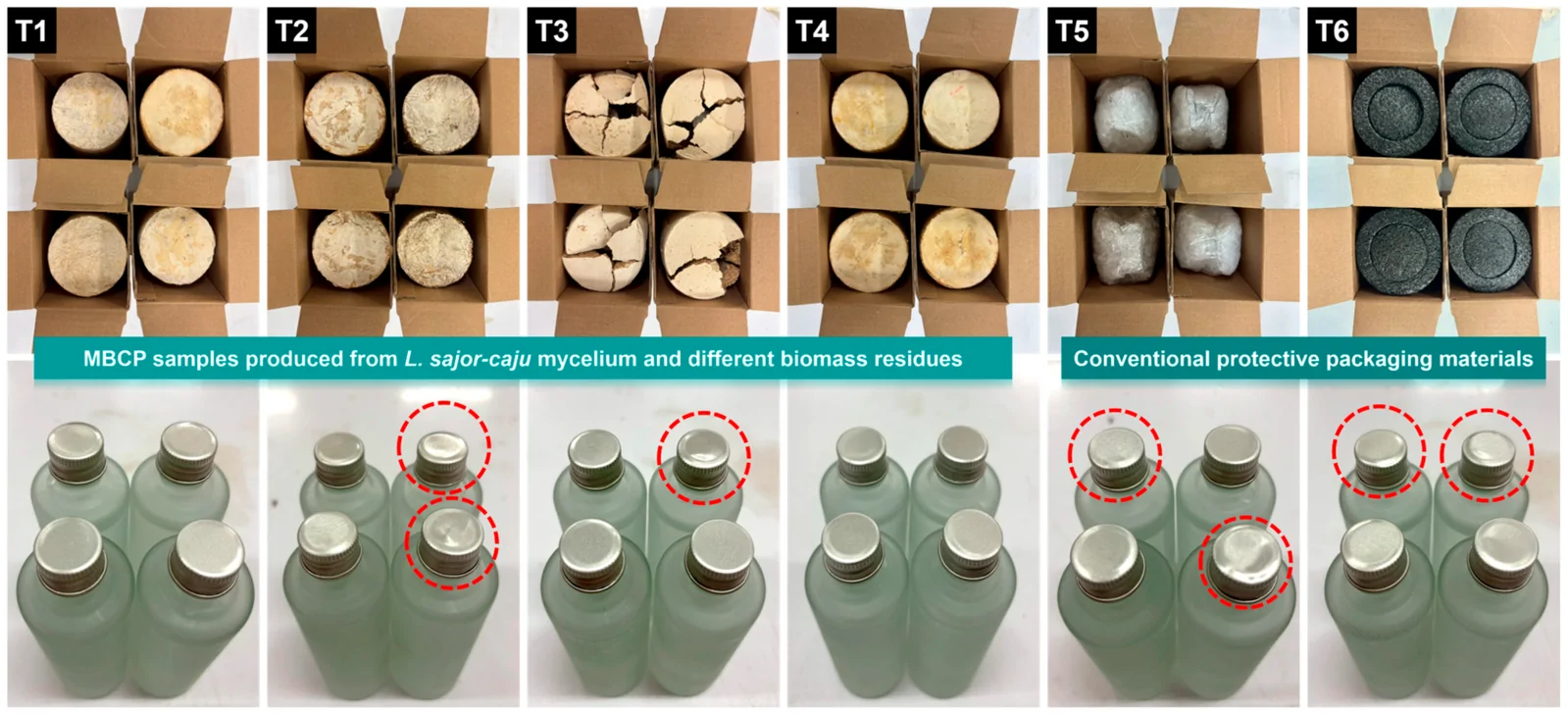
Representative damage to the developed MBCP and the associated blemishes on glass test bottles after the free-fall drop impact resistance test. Red circles indicate faulty lids. (T1) MBCP produced from corn husk; (T2) MBCP produced from rice straw; (T3) MBCP produced from sawdust; (T4) MBCP produced from sugarcane leaf; (T5) air bubble wrap; and (T6) EPE foam.

**Table 1:** The dimensional and geometric characteristics of the developed MBCP and comparable packaging materials.

Treatment	Thickness (mm)	Inner Diameter (mm)	Outer Diameter (mm)	Height (mm)	Depth (mm)	Volume (m^3^)	Geometry	Coverage Area (%)	Bottle and Packaging Gap (mm)
T1: MBCP produced from corn husk	7.4 ± 0.3	46.75 ± 0.5	60.4 ± 0.5	76.05 ± 1.6	149.5 ± 3.2	256.5 ± 5.7	Cylinder	100	26.5 ± 3.2
T2: MBCP produced from rice straw	7.4 ± 0.2	47.68 ± 0.7	60.2 ± 0.4	73.48 ± 1.0	144.7 ± 1.9	258.2 ± 8.2	Cylinder	100	21.7 ± 1.9
T3: MBCP produced from sawdust	8.3 ± 0.3	44.73 ± 0.4	61.8 ± 0.7	78.38 ± 0.9	154.5 ± 0.7	241.1 ± 4.7	Cylinder	100	30.5 ± 0.7
T4: MBCP produced from sugarcane leaf	7.6 ± 0.3	46.10 ± 0.1	60.9 ± 0.1	74.80 ± 1.4	147.3 ± 2.5	245.7 ± 4.6	Cylinder	100	24.3 ± 2.5
T5: Air bubble wrap	7.6 ± 0.1	43.03 ± 0.1	61.7 ± 0.4	75.20 ± 0.2	150.4 ± 0.5	218.6 ± 1.3	Roll sheet	100	27.4 ± 0.5
T6: EPE foam	9.5 ± 0.1	46.53 ± 0.1	63.4 ± 0.2	76.70 ± 0.9	151.9 ± 0.6	258.0 ± 1.2	Cylinder	100	28.8 ± 0.6

Note: The tested bottle was 41 mm in diameter, 123 mm in height, and 85 mL in capacity.

**Table 2:** Primary physical properties of MBCP developed from different biomass residues combined with *L. sajor-caju* mycelium.

Treatment	Volumetric Shrinkage (%)	Dried Weight (g)	Dried Density (kg/m^3^)
T1: MBCP produced from corn husk	15.29 ± 1.53 ^a^	29.26 ± 1.38 ^b^	243.18 ± 8.23 ^b^
T2: MBCP produced from rice straw	13.44 ± 1.17 ^a^	28.43 ± 1.86 ^bc^	216.49 ± 2.62 ^d^
T3: MBCP produced from sawdust	5.23 ± 3.24 ^b^	38.74 ± 2.35 ^a^	314.79 ± 3.91 ^a^
T4: MBCP produced from sugarcane leaf	5.55 ± 1.48 ^b^	26.32 ± 1.09 ^c^	229.80 ± 7.75 ^c^

Note: Data are presented as the mean ± standard deviation. Different letters within the same column indicate significant differences according to Duncan’s multiple range test at *p* < 0.05.

**Table 3:** Ultimate compressive strength of MBCP fabricated from different lignocellulosic biomass residues using *L. sajor-caju* mycelium.

Treatment	Ultimate Compressive Strength (MPa)
T1: MBCP produced from corn husk	0.43 ± 0.17 ^c^
T2: MBCP produced from rice straw	0.29 ± 0.08 ^c^
T3: MBCP produced from sawdust	0.91 ± 0.13 ^a^
T4: MBCP produced from sugarcane leaf	0.68 ± 0.11 ^b^

Note: Data are presented as the mean ± standard deviation. Different letters within the same column indicate significant differences according to Duncan’s multiple range test at *p* < 0.05.

**Table 4:** Comparison of the free-fall drop impact resistance of MBCP fabricated from different lignocellulosic biomass residues using *L. sajor-caju* mycelium with air bubble wrap and EPE foam.

Products and Specimens	Empty Weight (g)		Loaded Weight (g)	High Standard (m)	Leak		Faulty Lid		Crack		Deform		Scratch	
	M	B			M	B	M	B	M	B	M	B	M	B
T1: MBCP produced from corn husk	59.33 ± 1.92 ^b^	197.53 ± 0.33	287.75 ± 4.56 ^b^	0.76	–	no	–	no	no	no	no	no	yes	no
T2: MBCP produced from rice straw	56.55 ± 1.35 ^c^	197.43 ± 0.39	290.75 ± 4.16 ^b^	0.76	–	no	–	yes	yes	no	no	no	yes	yes
T3: MBCP produced from sawdust	79.25 ± 2.20 ^a^	197.53 ± 0.41	307.63 ± 6.96 ^a^	0.76	–	no	–	yes	yes	no	yes	no	yes	no
T4: MBCP produced from sugarcane leaf	51.65 ± 2.31 ^d^	197.43 ± 0.46	274.83 ± 5.76 ^c^	0.76	–	no	–	no	yes	no	no	no	yes	no
T5: Air bubble wrap	9.46 ± 0.16 ^e^	197.68 ± 0.50	235.33 ± 0.31 ^d^	0.76	–	no	–	yes	no	no	no	no	no	no
T6: EPE foam	5.15 ± 0.26 ^f^	197.60 ± 0.37	230.93 ± 0.67 ^d^	0.76	–	no	–	yes	no	no	no	no	no	no

Note: Data are presented as the mean ± standard deviation. Different letters within the same column indicate significant differences according to Duncan’s multiple range test at *p* < 0.05. M = mycelium-based cushioning specimens; B = glass bottle containing 80 mL of water. No = no damage observed; Yes = damage observed; − = this type of damage was not applicable or not recorded.

**Table 5:** Comparison of petroleum-derived packaging materials and developed MBCP according to sustainable packaging principles adapted from Chopra et al. [60] and Yunlun-pack [63].

Packaging	MBCP	Air Bubble Wrap	Plastic Foam
Biodegradable	✓	–	–
Minimum material usage	–	✓	✓
Form: Sheet	–	✓	✓
Generic packaging	–	✓	✓
Recyclable	✓	–	–
Made from waste	✓	–	–
Primary applications	Protective packaging, including custom-molded cushions, trays, corner guards, and inserts for electronics, glassware, cosmetics, and other fragile products.	Protective cushioning for fragile products during storage and transportation, offering shock absorption and protection against moisture, dust, abrasion, and impact.	Cushioning and protective packaging, temperature-controlled packaging, surface protection, automotive and medical components, and custom storage solutions.
Economic feasibility	Potential for low-cost raw materials by the use of biomass residues, but economic competition remains limited by the 21-day incubation period, energy demand for maintaining 30 °C, and labor-intensive molding. Process optimization and automation are therefore essential for scalable and cost-effective production.	High production efficiency and low unit cost due to rapid, highly automated production.	High production efficiency and low unit cost due to high-speed, automated processing.
Relative package mass	Approximately 5–10× conventional materials (refer to Table 3)	Refer to Table 3	Refer to Table 3
Finished-product cost	14.50 THB (USD 0.4391)	0.17 THB (USD 0.0052)	21.40 THB (USD 0.6420)

Note: The information on MBC packaging applications is based on and summarized from Camilleri et al. [16], Akromah et al. [35], and Alemu et al. [42]. ✓ = characteristic is present; − = characteristic is absent or not applicable. Finished-product costs are based on the materials and commercial products available in the study area and may differ across manufacturers, production scales, and markets.

## Data Availability

The original contributions presented in this study are included in the article. Further inquiries can be directed to the corresponding author.
